# The Politics of Radiation Protection

**DOI:** 10.1007/s00048-022-00332-z

**Published:** 2022-05-02

**Authors:** Maria Rentetzi

**Affiliations:** 1 Chair of Science, Technology and Gender Studies, Friedrich-Alexander-Universität, Werner von Siemens Strasse 61, 91052 Erlangen-Nürnberg, Germany; 2 Max Planck Institute for History of Science, Boltzmannstrasse 22, 14195 Berlin, Germany

**Keywords:** History of radiation protection, International organizations, IAEA, WHO, UN, South Korea, Mexico, Spain

The latest edition of the Basic Safety Standards, entitled the International Basic Safety Standards for Protection Against Ionizing Radiation and for the Safety of Radiation Sources, is the product of extensive global cooperation. The BSS are jointly established together with five other organizations, including the International Labour Organization and the World Health Organization. (González 1998: 2)

In 1998 the International Atomic Agency (IAEA) published a special issue of its *Bulletin* dedicated to the development of nuclear and radiation safety standards. As Abel González, the then director of the IAEA’s Division of Radiation and Waste Safety argued, the 1990s had seen the “emergence of […] an ‘international regime on nuclear and radiation safety’” (ibid.). In the wake of the Chernobyl nuclear accident, revisions of radiation safety standards and the introduction of the concept of safety culture were a response to a crisis situation in the nuclear industry. This new “international regime” concerning radiation protection and nuclear safety has been projected legally binding undertakings among member states, globally agreed-upon international safety standards, and provisions for facilitating the application of those standards.^1^ Yet the history of radiation protection and the development of radiation standards is far from a linear story of progress. It is more than just a story of scientific cooperation on an international level that requires interstate relations and assumes rigid national boundaries. Rather it asks for a broader conception of international relations, nuclear diplomacy (Ito & Rentetzi 2021; Rentetzi & Ito 2021), and the circulation of materials, knowledge, and expertise, all pointing to the role of international organizations and both national and international regulatory institutions. In short, I argue, it demands the use of international governance—the sum of institutions that coordinate transnational actors through not only traditional means such as legal tools and policies but also via scientific standards and materials—as an analytical framework.

This special issue focuses on those organizational structures, material resources, diplomatic practices, and national ideologies that facilitated the social and political shaping of the scientific field of radiation protection in the 1960s and 1970s.^2^ The authors collectively explore the nuclear regulatory system that the United Nations and, in particular, the World Health Organization (WHO) and the IAEA helped to shape. We also focus on some key national responses to the politics of radiation protection that techno-political organizations and nuclear industries were promoting. On the whole, we argue that radiation protection is not merely a technoscientific issue but a highly political, ideological, and—especially within the UN regulatory system—a multinational diplomatic endeavor that requires a transnational and interdisciplinary approach. This special issue unearths the peculiarities of the history of radiation protection in three national contexts (Mexico, Spain, and Korea) and for two UN international organizations (WHO and IAEA). Once radiation protection slipped from the hands of health physicists—the professionals who initially sought to devise an organization exclusively concerned with radiation protection during the Manhattan Project —it became a highly controversial political and diplomatic issue. Let us briefly review this trajectory.

Historians have highlighted scientists’ struggles to define the appropriate unit of radiation and their long-standing efforts to invent suitable measurement devices and improve instrumentation, to detect and agree on the effects of radiation on biological systems, to identify the acceptable level of risk with regard to radiation exposure and to agree on safety radiation standards in both medicine and industry. The scientific controversies that emerged through the above processes reflect the complexity and messiness of scientists’ collaborative production of what eventually comes to be considered as reliable knowledge. They also reveal the powerful role of those scientific institutions that assumed the task of regulating radiation and enforcing acceptable standards.^3^

Until 1934, the recommendations on radiation protection referred to medical operating procedures for the use of X‑rays and radium, with a particular focus on equipment design, shielding from radiation sources, and shielding of rooms and enclosures with lead. In parallel, research into the genetic effects of radiation exposure was getting underway: Hermann Joseph Muller’s genetic research highlighted the pressing need to better understand the biological effects of radiation and to regulate exposure to it.^4^ This required a wholesale transformation of attitudes towards radiation dangers and major changes in laboratory culture. In the interwar atomic research laboratories, radiation hazards had often been ignored altogether. Radiation protection became an issue of concern only during World War II and came to a peak with the establishment of the IAEA in 1957 (Rentetzi 2017).

As powerful institutions started to take up the elaboration of radiation standards and protection practices, as well as their legal requirements, the field of radiation protection as a whole was characterized by disagreement and controversy. Wartime was marked by the US Atomic Energy Commission’s (AEC) attempts to downplay the consequences of exposure to radiation and by the classified status of research on the topic. The studies that the AEC sponsored were often designed to prove that it was relatively harmless for humans to work at facilities such as Oak Ridge, a major national nuclear facility for the Manhattan Project, irrespective of the costs paid by humans used as experimental subjects (Olwell 2004; Walker 1989; Walker 1994). As Linda Richards reminds us in this issue, these were “the brutal experimental origins of health physics.”

In the aftermath of the bombings of Hiroshima and Nagasaki there was a radical change in the conceptualization of radiation safety as it shifted from being an issue of secondary concern to radiologists and radiological physicists to a major task and the subject of a new discipline. The broad public concern about the effects of radiation, as well as the rapid development and adoption of new medical technologies (such as the radioisotope teletherapy units) and the emergence of the nuclear industry posed numerous challenges in the field of radiation protection.^5^ To address these issues, the US Advisory Committee on X‑Ray and Radium Protection was reassembled as the National Committee on Radiation Protection and embarked on several studies in order to revise radiation standards. Also in the US, the Health Physics Society was formed in 1955, with a primary interest in radiation protection. Although the American Industrial Hygiene Association (AIHA) had been founded as early as 1939, it covered only some aspects of the occupational health risks related to radiation exposures (Kathren 1978). But what proved to be most challenging was the fact that the issue of radiation protection forced governments to sit at the same table and negotiate. To do so, scientists, diplomats, technical experts, insurers, and lawyers had to share their expertise and skills (Mitchell 2021; Kyrtsis & Rentetzi 2021).

In early spring 1953 experts in radiation protection representing their countries—the US, Canada, and England—met at Arden House in Harriman, on the outskirts of New York. The meeting resulted in agreement on some final recommendations for new radiation protection standards. This was the third in a series of conferences that became known as the Tripartite Conferences on Radiation Protection. The first had taken place in September 1949 at Chalk River, Ontario, the site of the Anglo-Canadian wartime nuclear research project, the second the following summer at Harwell, the UK’s new atomic research facility. The tripartite initiative resulted from informal contacts between scientists researching ionizing radiation. At stake in Harriman was the harmonization of protection practices and close technical cooperation among the three countries. This meeting symbolized the governments’ recognition of the dangers ionizing radiation posed to human health and served as an acknowledgment that work in the burgeoning nuclear industries and in military laboratories could not continue without revised radiation protection standards and an agreed system of international regulation (Taylor 1953; see also Lambert 1990).

In this period safety standards issued by national and international regulatory institutions were heavily based on the study of bomb survivors, limiting their ability to account for low levels of radiation exposure (Lindee 2016). The mass quantities of radioactive materials and new types of radiation enforced new approaches in the field and created the space for the international regulation of radiation risks (Boudia 2007; de Chadarevian 2015). Gradually it was the IAEA, primarily a political and diplomatic organization, that took the lead in radiation protection, displacing organizations such as the WHO, the International Labour Organization (ILO), and the International Commission on Radiological Protection (ICRP). At the same time the IAEA played a crucial role in an exceptionally broad range of scientific and political matters that often conflicted with international attempts to reduce the use of nuclear energy, control the industry, and protect humans and the environment from ionizing radiation.^6^

The special issue begins with two essays that question the role of major UN organizations in framing radiation protection on a global level. Linda Richards shows how the WHO’s approach of conceptualizing radiation protection as an institutional responsibility and a human right to health failed due to the preeminence of American risk models of radiation regulation that have dominated the discussion. Her paper reveals the untold story of how Brock Chisholm, the WHO’s first Director General, became the leading advocate of a new concept of radiation protection as a human right to health, encompassing physical, mental, and social well-being. Despite his activities, the actors who set the agenda and actually defined radiation protection were the scientists working for the US Atomic Energy Commission. As the US was the major funder of the WHO, it was able to discipline the experts of the international organization. Based on extensive archival research, Richards also touches on the conflicts between the newly established IAEA and the WHO over radiation standards and predominance in the field. Maria Rentetzi questions the politics of radiation dosimetry by focusing on the beginnings of the IAEA’s radiation dose intercomparison program that took shape during the early 1960s. In collaboration with the WHO, the agency tried to standardize dosimetry on a global level. Introducing the concept of a *global experiment*, Rentetzi shows that the IAEA’s agenda included not only technical assistance to member states but presupposed research and thus a reconceptualization of scientific experimentation. What started as an intercomparison of radiation doses among a few European institutions in the early 1960s ended up being an immense open-ended experimental procedure that involved participating laboratories from across the world. Through a step-by-step analysis of the development of IAEA’s dosimetry, Rentetzi argues that radiation protection has never been simply a technical issue, but instead it also required multinational diplomacy and the institutional system of the UN in order to be standardized across the globe.

The next two essays address the development of nuclear safety and health physics in different national contexts, highlighting the ways that knowledge and practices move across national and international institutions. Focusing on Spain, Ana Romero de Pablos explores how the circulation of nuclear artifacts deeply transforms knowledge about their use, scientific practices, and industrial management—while also reshaping notions of radiation safety and creating an entanglement of the political and diplomatic with the technical and scientific realm. In 1964, a Westinghouse nuclear power reactor arrived in Spain, bringing along regulations and experimental practices related to radiation safety and protection that were new to the local community of scientists and engineers. Romero de Pablos’s paper gives us a glimpse of how international organizations such as the IAEA affect local nuclear practices, how political and economic considerations are decisive over technological solutions, and how Spain’s first nuclear power plant prompted a stream of legal, economic, and scientific transformations in relation to nuclear safety. This study is greatly benefited by unique and rare material from the archives of Tecnatom, the engineering company that coordinated the construction of the plant. John DiMoia highlights the power of knowledge and practice transfer in the field of radiation protection in a radically different context, that of postwar South Korea. Challenging the American-dominated institutional history of Korea, DiMoia argues that Koreans crafted a version of health physics at the intersection of medicine and agriculture, and in a way that suited both their colonial past and their cooperation with new American counterparts. His provocative essay suggests that by exploring the Korean version of radiation protection, we can see the rise of a cluster of related interests, including environmental science and the effects of industrialization.

The special issue ends with an essay that examines the ways in which the technical collaboration between national and international institutions was deeply political *and* diplomatic, shaping research agendas on radiation protection. Ana Barahona offers an analysis of the development of Mexico’s radiobiology, including radiation protection, as a complex field that required the support of the IAEA and of an extended local network of scientists and institutions. She traces the development of radiation dosimetry and protection programs in the country, which were both strongly supported by the IAEA. In her detailed study, Barahona unravels entangled scientific interactions on local and international levels, shedding light on the inextricable relation between radiobiology research, nuclear industrial establishments, and radiation protection. In the end, what unites all the papers in the special issue is their focus on the role of UN international organizations such as WHO and IAEA in shaping local contexts and imposing regulatory frames during periods in which the international organizations were struggling to create their own niche in the field.

Today the renewed interest in nuclear power plants and the use of advanced nuclear medical technologies pose new challenges to the field of radiation protection. Scientists have only recently realized that radiation protection is not a purely scientific matter, but is also a social and political concern. In February 2016, the IAEA organized an international conference on the “Human and Organizational Aspects of Assuring Nuclear Safety” that focused mainly on nuclear power plants. It was the first time that the agency placed such an emphasis on the human factors affecting the safety culture of the nuclear industry and called for a reconceptualization of the term. In his introductory remarks the then Director General Yukiya Amano urged participants to reflect upon the lessons that had been learned over the last 30 years, that is, since the Chernobyl disaster. In addition, the IAEA’s 2015 Director General’s Report on the Fukushima Daiichi Accident left no doubts that human and organizational factors played a significant role in the management of the nuclear disaster following the earthquake and the subsequent tsunami in Japan. As Amano argued, “[a] major factor that contributed to the accident was the widespread assumption in Japan that its nuclear power plants were so safe that an accident of this magnitude was simply unthinkable” (IAEA 2015: Foreword).

Besides the IAEA, other regulatory agencies and stakeholders have recently noticed that safety is not an issue that should be left to nuclear scientists and engineers alone. In 2012 the International Radiation Protection Association (IRPA) held its international congress in Glasgow under the overarching theme “Living with Radiation—Engaging with Society.” As the organizers argued, “[t]he management of protection will always involve social decisions and choices.” William Magwood, Director-General of the Nuclear Energy Agency (NEA), a specialized agency within the OECD, wrote in his 2016 report on the Fukushima accident that “we must address the human aspects of safety, such as ensuring effective safety cultures for both operators and regulators and continuing to learn from safety research, including through the NEA’s international joint research projects.” And in the sector of nuclear medicine, the “Bonn Call for Action,” a joint position statement published by the IAEA and the WHO in 2012, argued for a holistic approach to the problem of radiation protection and called for the inclusion of actors from civil society as well. One of the call’s major proposed actions is to improve radiation safety culture in health care.^7^

At the same time, over the last decade the nuclear industry has been largely left behind by the booming demand for clean energy. Especially after the Fukushima accident and due to major concerns over radiation protection among the general public, more reactors were permanently shut down than built. It seems that investors have abandoned nuclear technology—not only because of the new public awareness, but also due to high costs and delays in construction. Obviously, given the lack of new investments, the average age of the world’s reactors is increasing, requiring ever more resources dedicated to maintenance and safety. According to the 2021 World Nuclear Industry Status Report, out of the 438 reactors in operation globally in 2002, only 415 were operating in mid-2020, the lowest number over the last 30 years. Also, out of the 52 new plants under construction at the time of the report, at least 33 were behind schedule, mainly because of the luck of investments. Not a single project came up in the first half of 2020 and global nuclear investment dropped drastically while resources spent on wind and solar energy are increasing. Only China continued to invest in nuclear energy, bypassing France to become second after the U.S. in nuclear energy production. In the foreword of the report, Naoto Kan, Japan’s prime minister at the time of the Fukushima disaster, proclaimed that, “I now believe that the time has come for Japan and the world to end its reliance on nuclear power” (*World Nuclear Industry Status Report* 2021: 15).

Yet, late in 2021, the European Commission surprised the world by publishing a draft proposal that classifies nuclear energy as climate-friendly, a taxonomy that aims to revive the nuclear industry. And the EU is not alone in promoting nuclear energy as sustainable. In addressing the 2020 United Nations General Assembly, IAEA’s Director General Rafael Mariano Grossi confirmed that “nuclear power is part of the solution to the climate crisis.”^8^ The IAEA, the organization that promotes itself as the world’s center for nuclear cooperation, is indeed encouraging countries to consider building smaller, more modular variants of reactors.

Critical voices, however, remind us that the nuclear industry is not and cannot be sustainable given that nuclear power plants are uniquely threatened by climate change—just think of the Fukushima accident – or more recently, by war (Brown & Solomon 2022). In addition, the uranium needed for the operation of nuclear plants is finite. A recent joint IAEA and NEA report foresees that demand for the material will increase especially in the East Asia region and will be adequate until 2040 (*Uranium 2020* 2020). Uranium extraction requires the advancement of mining and processing technologies while societal expectations related to environmental protection and workers’ safety require stricter radiation protection measures. According to Grossi, “[t]he great benefits of nuclear technologies are sustainable only if they are used safely and securely.”^9^

However, technological and scientific standards alone have not been sufficient in protecting humans and the environment from radiation. This special issue hopes to keep up the momentum created by the above agencies and institutions and, in addition, offer a historical and critical perspective on why human and social risks have been underrated. We bring together scholars working on the history of the nuclear sciences and the role of international organizations in shaping policies, promoting specific lines of research and technologies, and producing standards—while arguing that humanities and social sciences have much to contribute to the current debate on radiation protection.

## Footnotes

1 For the sake of consistency in the use of terminology among scholars coming from different disciplines, I adopt the IAEA’s terminology with regard to the meaning of radiation protection and nuclear safety. Radiation protection refers to the “protection of people and the environment against radiation risks.” Nuclear safety denotes the safety of “facilities and activities that give rise to radiation risks.” Put together, “‘protection and safety’ […] encompasses the safety of nuclear installations, radiation safety, the safety of radioactive waste management and safety in the transport of radioactive material.” Obviously, the two terms are closely intertwined. Since safety is primarily concerned with control over sources it contributes towards protection (IAEA Safety Glossary 2019: 2).

2 This issue is the belated result of a workshop entitled “Claiming Authority, Producing Standards: IAEA and the History of Radiation Protection” that I organized jointly with Martin Kusch in Vienna in 2016. Besides the authors of this issue, I would like to thank all the participants including Martin Kusch, Prakash Hande, Fabian Lüscher, Michael Hutter, E. Jerry Jessee, John Downer, Alison Kraft, Laura Sembritzki, María Jesús Santesmases, Kate Brown, Angela Creager, Jacob Hamblin, and Soraya de Chadarevian.

3 See, for instance, Asimov & Dobzhansky (1966); Caufield (1989); Kevles (1997); Preston (2005); Rentetzi (2004); Rentetzi (2022); Serwer (1976); Taylor (1979); Walker & Wellock (2010); Witthemore (1986); see also special issue of *Health Physics *(2005).

4 Campos (2015); Carlson (1981); Hamblin (2007); Lindee (2013); Barahona in this issue.

5 Beatty (1993); Creager (2013); Creager (2015); Howell (1995); Kevles (1997); Mazuzan & Walker (1984); Mould (1993); Walker (2000).

6 Fischer (1997); Forland (1997); Rentetzi (2017); Roehrlich (2016); Scheinman (1987).

7 See http://www.irpa.net/page.asp?id=54516; https://www.oecd-nea.org/news/2016/2016-01.html; https://www.iaea.org/sites/default/files/17/12/bonn-call-for-action.pdf (13.02.2022).

8 https://www.iaea.org/newscenter/statements/director-generals-statement-to-the-75th-regular-session-of-the-united-nations-general-assembly.

9 Ibid.
